# Detection and size quantification of pulmonary nodules in ultralow-dose versus regular-dose CT: a comparative study in COPD patients

**DOI:** 10.1259/bjr.20220709

**Published:** 2023-02-20

**Authors:** Daiwei Han, Jiali Cai, Anne Heus, Marjolein Heuvelmans, Kai Imkamp, Monique Dorrius, Gert-Jan Pelgrim, Gonda de Jonge, Matthijs Oudkerk, Maarten van den Berge, Rozemarijn Vliegenthart

**Affiliations:** 1 Department of Radiology, University of Groningen, University Medical Center Groningen, Groningen, The Netherlands; 2 Department of Epidemiology, University of Groningen, University Medical Center Groningen, Groningen, The Netherlands; 3 Department of Radiology, Medisch Spectrum Twente, Enschede, The Netherlands; 4 Department of Pulmonology, Medisch Spectrum Twente, Enschede, The Netherlands; 5 Department of Pulmonology, University of Groningen, University Medical Center Groningen, Groningen Research Institute for Asthma and COPD, Groningen, The Netherlands; 6 Institute for Diagnostic Accuracy Research B.V., Groningen, The Netherlands; 7 University of Groningen, Groningen, The Netherlands

## Abstract

**Objective:**

To evaluate detectability and semi-automatic diameter and volume measurements of pulmonary nodules in ultralow-dose CT (ULDCT) *vs* regular-dose CT (RDCT).

**Methods:**

Fifty patients with chronic obstructive pulmonary disease (COPD) underwent RDCT on 64-multidetector CT (120 kV, filtered back projection), and ULDCT on third-generation dual source CT (100 kV with tin filter, advanced modeled iterative reconstruction). One radiologist evaluated the presence of nodules on both scans in random order, with discrepancies judged by two independent radiologists and consensus reading. Sensitivity of nodule detection on RDCT and ULDCT was compared to reader consensus. Systematic error in semi-automatically derived diameter and volume, and 95% limits of agreement (LoA) were evaluated. Nodule classification was compared by κ statistics.

**Results:**

ULDCT resulted in 83.1% (95% CI: 81.0–85.2) dose reduction compared to RDCT (*p* < 0.001). 45 nodules were present, with diameter range 4.0–25.3 mm and volume range 16.0–4483.0 mm^3^. Detection sensitivity was non-significant (*p* = 0.503) between RDCT 88.8% (95% CI: 76.0–96.3) and ULDCT 95.5% (95% CI: 84.9–99.5). No systematic bias in diameter measurements (median difference: −0.2 mm) or volumetry (median difference: −6 mm^3^) was found for ULDCT compared to RDCT. The 95% LoA for diameter and volume measurements were ±3.0 mm and ±33.5%, respectively. κ value for nodule classification was 0.852 for diameter measurements and 0.930 for volumetry.

**Conclusion:**

ULDCT based on Sn100 kV enables comparable detectability of solid pulmonary nodules in COPD patients, at 83% reduced radiation dose compared to RDCT, without relevant difference in nodule measurement and size classification.

**Advances in knowledge:**

Pulmonary nodule detectability and measurements in ULDCT are comparable to RDCT.

## Introduction

In view of the promising results from the NELSON trial,^1^ the implementation of lung cancer screening using CT is being considered across Europe.^2–4^

With a potentially massive increase in the number of chest CT scans worldwide, the potential radiation related health risks from lung cancer screening have become a major concern. While evidence in support or against the use of a linear no-threshold dose–response model^5^ for estimating cancer risk from low-dose radiation is lacking,^6,7^ the debate has driven the radiation dose in medical imaging to be set “as low as reasonably achievable”.^8^ The optimization of radiation dose in lung cancer screening using CT has become of major interest due to the accumulation of radiation exposure from short-term and annual follow-up examinations.

Currently, several techniques can be used to reduce the radiation dose, which include the optimization of X-ray tube voltage, reduction of X-ray tube current, automated dose modulation, and more recently introduced tin filtration.^9^ Yet, radiation dose reduction causes degradation of image quality, which is seen as an increase in image noise. Various iterative reconstruction (IR) methods and image processing techniques can be utilized to reduce image noise while possibly trading-off image detail by smoothing the image.^10^

While the evaluation of tin filtration and Advanced Modeled Iterative Reconstruction (ADMIRE) in previous chest CT phantom studies, on lung nodule detection have yielded favorable results,^9–12^ the knowledge of their application *in vivo* is limited, especially for how the smoothing of image may affect semi-automatic volumetry of pulmonary nodules.The purpose of this study was to investigate the accuracy of ultralow-dose CT (ULDCT) with tin filtration for detection and semi-automatic volumetry of pulmonary nodules in a patient cohort, compared to regular-dose CT (RDCT).

## Methods and materials

### Study population

The current analysis is a substudy of a treatment study in chronic obstructive pulmonary disease (COPD) patients.^13^ This prospective, single-center study was approved by the local medical ethics committee, and each participant gave his or her informed consent. The inclusion criteria for the treatment study were: age between 40 and 80 years, smoking history of ≥10 pack years, COPD with a forced expiratory volume (FEV1) <80% predicted with or without inhaled corticosteroids, and at least one COPD exacerbation for which oral prednisolone was prescribed 2 years prior to the inclusion of the study. Based on the FEV1 results, each patient was classified into a Global Initiative for Obstructive Lung Disease (GOLD) category.^14^ Exclusion criteria were history of asthma, recent exacerbation, or respiratory tract infection within a month prior to study enrollment, and females with childbearing potential. For the current substudy, participants underwent an ULDCT scan on top of the RDCT scan that was part of the treatment study. From February 2018 to July 2018, 50 patients were enrolled in this substudy. Sample size was based on the cohort size of prior articles comparing emphysema in ULDCT to non-contrast chest CT. The scanning procedure of both RDCT and ULDCT was explained to the participants.

### CT imaging

Inspiratory chest CT scanning was performed on a 64-multidetector CT system (Somatom Definition AS, Siemens Healthineers, Erlangen, Germany) and on a third-generation dual-source CT system (Somatom Force, Siemens Healthineers, Erlangen, Germany) with a maximum of 30 min between the two acquisitions, with random order per patient. Each participant received the bronchodilator Salbutamol (100 µg, inhaled) before CT scanning. As reference, a regular-dose CT (RDCT) scan was acquired using the 64-multidetector CT system at tube voltage 120 kVp, and a fixed tube current time product of 40 mAs, in line with the Quantitative Imaging Biomarkers Alliance (QIBA) profile.^15^ Third-generation dual-source CT was used to acquire an ultralow-dose chest CT scan at 100 kVp with 0.6 mm tin filter for spectral shaping, and tube current modulation (CAREDose4D, Siemens Healthcare) with tube current time product of 70 mAs (reference mAs). The purpose of tube current modulation in ULDCT scanning is to maintain constant signal-to-noise ratio across different sections of the body.^9,11,16^ Both CT scans were reconstructed with a field of view of 350 mm and slice thickness of 1.0 mm, at 0.7 mm increment. For nodule detection and semi-automatic volumetry, medium smooth kernel, B30f and Br40, were reconstructed for 64-multidetector CT and third-generation dual-source CT, respectively.

### Image analysis

To reduce effect of learning curve and recall bias, the first reader (MD, radiologist with 12 years of experience in chest imaging) was blinded from patient information. Additionally, RDCT and ULDCT patient scans were randomized and presented to the reader in an alternating manner within 2 weeks on four different days. The CT reconstructions were evaluated using image analysis workflow software MM Oncology (Syngo.via, v. VB10A, Siemens, Erlangen, Germany). The first reader was allowed to freely use maximum intensity projection and multiplanar reformation for nodule detection. Nodules of at least 4 mm in maximum transverse diameter were included in this study. To avoid missing nodules due to possible fluctuations in nodule measurement on RDCT and ULDCT, all nodules greater than 2 mm by visual estimation were first marked with a circular region of interest (ROI) by the first reader. Subsequently, diameter and volume of all marked nodules were measured by a researcher (DH) by point and click method using a semi-automatic volumetry function in the MM Oncology software, which also provided maximum transverse diameter of the nodule. Unsatisfactory segmentation of a nodule due to attachment was manually corrected using the modify function. Nodules that were marked with ROI on RDCT and ULDCT were paired based on anatomical landmarks. Nodules with at least 4 mm in transverse diameter measured on RDCT were selected for further analysis.

Discrepancy in nodule detection between RDCT and ULDCT by the first reader were reviewed independently by two radiologists (RV and GdJ) with 15 years and 12 years of experience in chest imaging, respectively. Disagreements were resolved through a consensus meeting. Nodules that were missed on either RDCT or ULDCT were retrospectively detected and measured to be included in the nodule measurement comparison. Maximum transverse diameter measured on RDCT of at least 4 mm determined inclusion for further analysis.

Image noise was assessed by drawing an ROI in the lumen of trachea, 1 cm above the bifurcation of the trachea. The standard deviation of Hounsfield unit (HU) of 1 cm in diameter was used as measure of noise. The ROIs were drawn by a trained researcher (DH) on both RDCT and ULDCT data sets.

Radiation dose parameters for both LDCT and ULDCT, including computed tomography dose index (CTDI_vol_) and dose–length product (DLP), were retrieved from the dose report of each CT scan. The effective dose was calculated by multiplying DLP with the chest conversion coefficient (*k* = 0.014 mSv/mGy*cm).^17^

### Statistical analysis

Nodule detection was compared between ULDCT and RDCT using reader consensus as the reference standard for nodule detection. Residual plot and 95% limits of agreement (95% LoA) were used to analyze the impact of ULDCT on nodule diameter and volume measurements compared to RDCT as reference standard. For the purpose of comparability with the findings from literature,^18^ the 95% LoA was analyzed using absolute values and percentage values for diameter and volume measurements, respectively. McNemar’s test was used to compare the sensitivity of nodule detection between ULDCT and RDCT. Wilcoxon signed rank test was used for the analysis of systematic error in nodule size quantification Cohen’s κ was used for the analysis of agreement in nodule size classification in diameter (<5 mm, 5 to <10 mm, and ≥10 mm) and volume (<100 mm^3^, 100 to <300 mm^3^, ≥300 mm^3^), in line with recommendations from the European Position Statement on Lung Cancer Screening.^2^ The difference in image noise between RDCT and ULDCT was evaluated by comparing the median standard deviation of the Hounsfield unit, and tested using Wilcoxon signed rank test. Normality of data distribution was determined using Kolmogorov–Smirnov test. Normally distributed variables were presented as mean and standard deviation (SD), non-normally distributed variables were presented as median and interquartile range (IQR). A *p*-value < 0.05 was considered statistically significant. Statistical analysis was performed using SPSS v. 23 (SPSS, IBM, New York).

## Results

### Demographics

Mean age of participants was 65.4 ± 7.3 years, range 50–82 years. Of the study participants, 34/50 were male (68%). The median body mass index (BMI) was 27.1 kg/m^2^ (IQR: 25.0–31.3), and the median number of smoking pack-years was 37.5 (IQR: 27.8–59.3). Based on the (GOLD) classification for COPD, 31/50 (62%) of patients had GOLD Stage 2, 14/50 (28%) GOLD 3, and 5/50 (10%) GOLD 4.

The median difference in the standard deviation in HU between RDCT and ULDCT was 13.8 HU (*p* < 0.001) (Table 1). The estimated effective dose reduction was 83.1% (95%CI: 81.0–85.2) for ULDCT compared to RDCT.

**Table 1.** Overview of CT parameters for RDCT and ULDCT

	RDCT (95% CI)	ULDCT (95% CI)	*p*-valuea
Standard deviation of HU (HU)	18.8 (18.4–22.3)	33.7 (32.4–35.2)	<0.001
CTDIvol (mGy)	3.0b	0.4 (0.3–0.5)	<0.001
DLP (mGy*cm)	104.6 (102.0–107.4)	16.6 (15.4–19.6)	<0.001
Estimated effective dose (mSv)	1.5 (1.4–1.5)	0.2 (0.2–0.3)	<0.001

CTDIvol = CT dose index volume, DLP = dose–length product; HU = Hounsfield unit,RDCT = regular-dose CT, ULDCT = ultralow-dose CT.

Numbers presented are in median.

a Wilcoxon signed-rank test.

b Constant.

### Nodule detection

**Figure 1.**
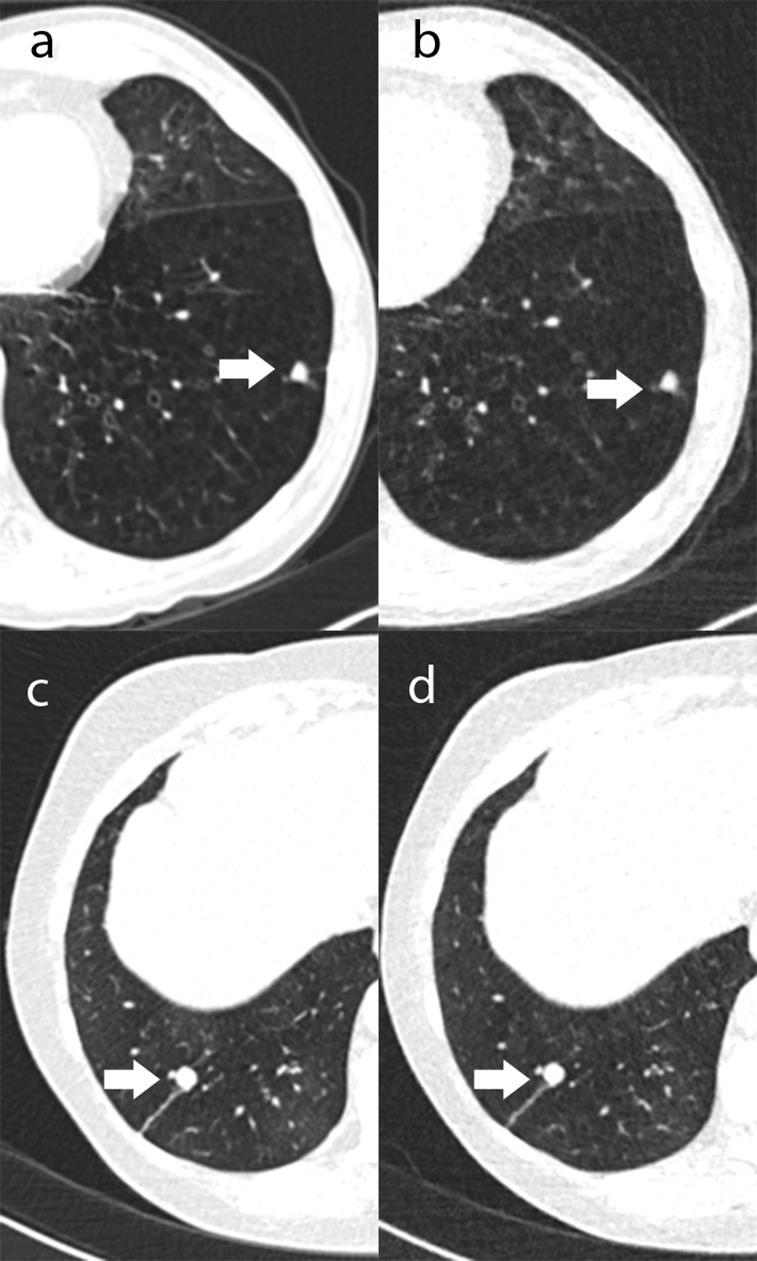
Transverse thorax CT images with true-positive nodules found on both ULDCT and RDCT from two patients. (**a**) and (**b**) are RDCT and ULDCT, respectively. (**c**) and (**d**) are RDCT and ULDCT, respectively. RDCT, response-dose CT; ULDCT, ultralow-dose CT.

**Figure 2.**
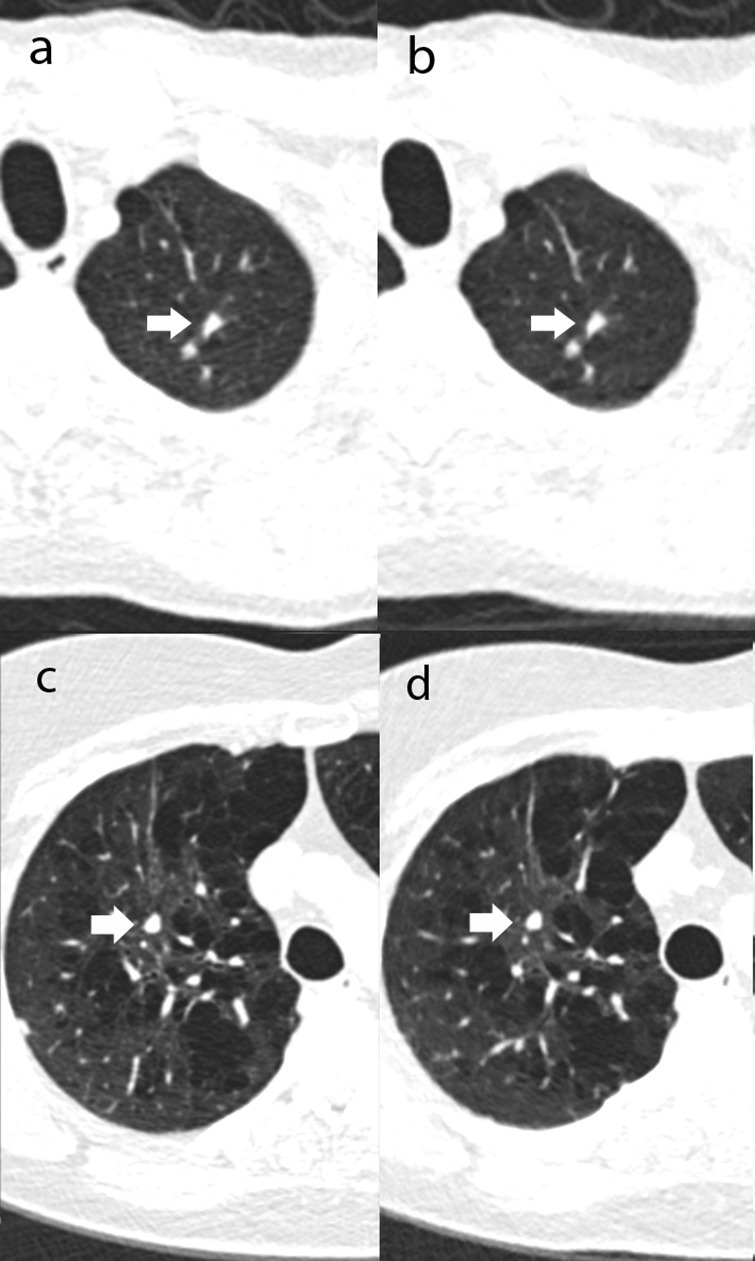
Transverse thorax CT images with nodules missed on ULDCT but found on RDCT from two patients. (**a**) and (**c**) are both RDCT. (**b**) and (**d**) are both ULDCT, assumed to be missed due to close proximity with apical lung fibrosis, and with bronchovascular bundle, respectively. RDCT, response-dose CT; ULDCT, ultralow-dose CT.

On the RDCT images, the first reader found a total of 45 solid nodules. On the ULDCT images, 49 solid nodules were found. There were a total of 57 unique nodules found on the RDCT and ULDCT scans, of which 37/57 nodules (64.9%) were found on both the RDCT and the ULDCT scan (Figure 1); these were considered as pulmonary nodules by default. From the 20/57 nodules that were missed on either RDCT^12^ or ULDCT,^8^ the consensus panel excluded seven nodules from RDCT and six nodules from ULDCT because they were considered false positive nodules, such as pleural thickening or fibrosis. Five nodules (11.4%) were missed on the RDCT scan, whereas two nodules (4.5%) were missed on the ULDCT scan (Figure 2). Ultimately, 44 nodules were agreed to be true positive nodules by reader consensus. The sensitivity for nodule detection for RDCT was 88.6% (95% CI: 75.4–96.2) and for ULDCT 95.5% (95% CI: 84.5–99.4), with a non-significant difference (*p* = 0.453). Nodules that were missed on either RDCT or ULDCT were all found in retrospect and subsequently included in size measurement comparison.

### Nodule diameter and volume measurements

**Figure 3.**
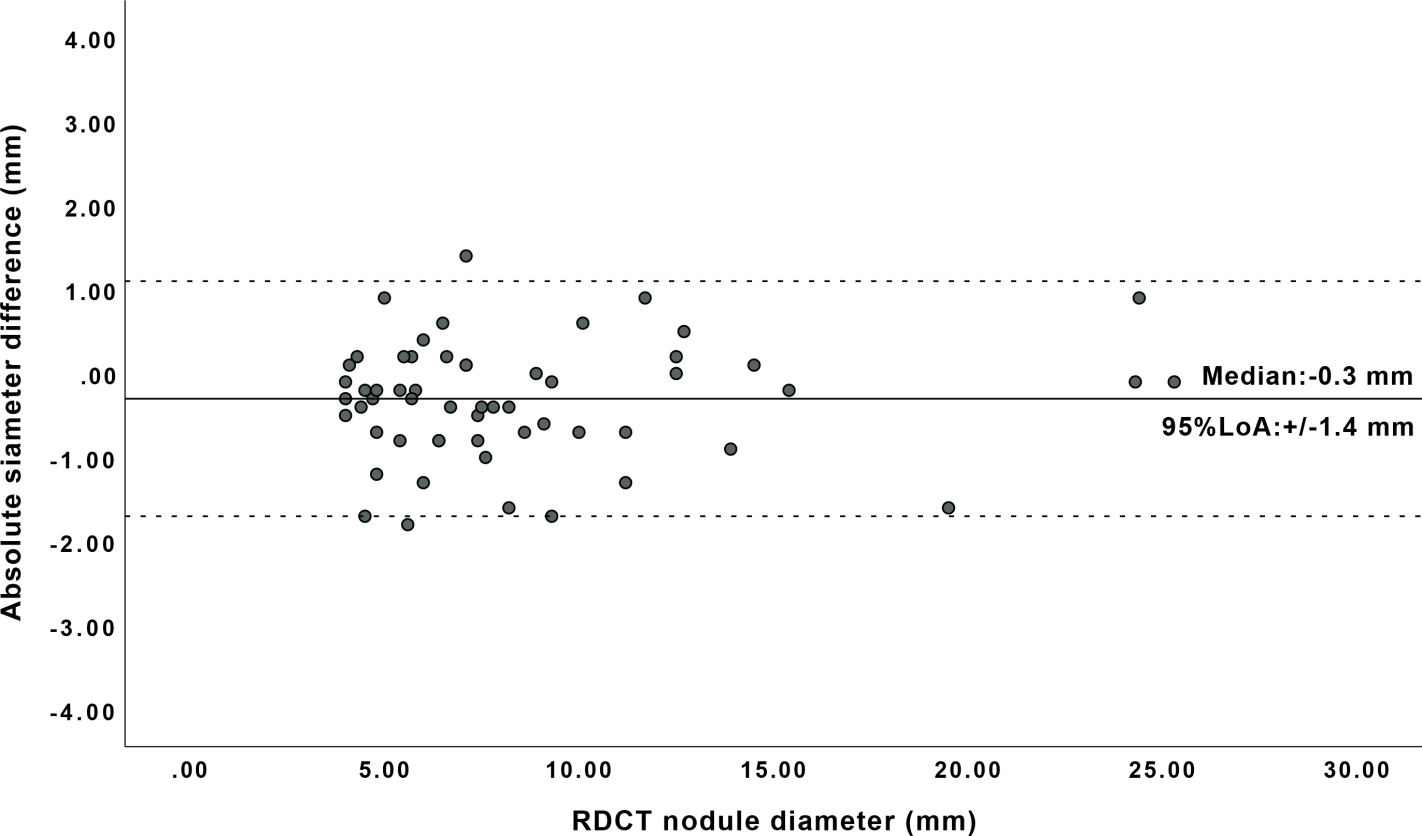
Residual plot for the semi-automatic diameter measurement of 44 nodules found in RDCT and ULDCT. RDCT, response-dose CT; ULDCT, ultralow-dose CT.

Two spiculated nodules in both RDCT and ULDCT required manual adjustment in segmentation. Both diameter and volume measurements of nodules were found to be non-normally distributed. The median diameter of all (true positive) nodules on RDCT was 7.3 mm (IQR: 5.6–10.9), and the median diameter on ULDCT was 7.0 mm (IQR: 5.5–10.5). The median nodule volume of nodules on RDCT was 106 mm^3^ (IQR: 48.8–229.8) and the median volume of nodules on ULDCT was 94 mm^3^ (IQR: 39.5–250.8). Significant difference in diameter [median difference: −0.2 mm (IQR: −0.7–0.2), *p* = 0.033) and volume [median difference: −6 mm^3^ (IQR:−12.3–4.0), *p* = 0.026) was found for ULDCT when compared to RDCT. The 95% LoA in diameter measurement was ±1.4 mm (Figure 3).

**Figure 4.**
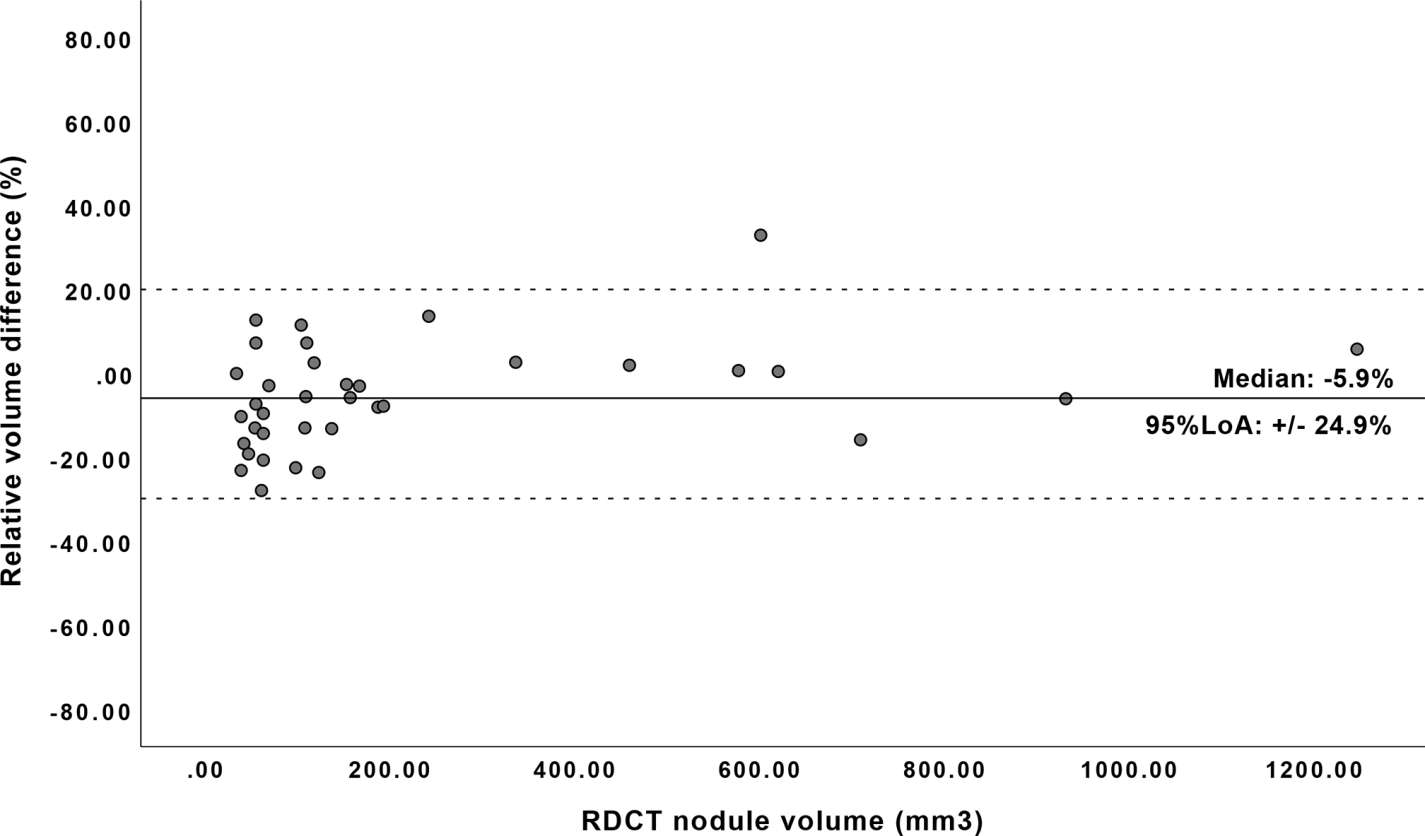
Residual plot for the semi-automatic volumetry of 38 nodules ≥ 30 mm^3^ found in RDCT and ULDCT. *Two nodules of above 2000 mm^3^ in size are not shown in the figure above. LoA, limits of agreement; RDCT, response-dose CT; ULDCT, ultralow-dose CT.

95% LoA in nodule volumetry was ±31.8% for all nodules. For nodules of at least 30 mm^3^ (38 nodules), the differences in volumetry between RDCT and ULCT were less pronounced with a 95% LoA of ±24.9% (Figure 4). For diameter nodule classification, agreement based on κ value was 0.847. On ULDCT, 4/44 (9.1%) of the nodules were classified into a lower size category compared to RDCT (Table 2). κ analysis showed excellent agreement in nodule volume classification between ULDCT and RDCT (*k* = 0.927); 2/44 (4.5%) nodules were classified into a lower size category on ULDCT compared to RDCT (Table 3).

**Table 2.** Crosstab for nodule diameter classification

	RDCT				
ULDCT	Diameter (mm)	<5	5 to <10	≥10	Total
	<5	7 (16)	2 (4)	0 (0)	9 (19)
	5 to <10	0 (0)	22 (49)	2 (4)	24 (53)
	≥10	0 (0)	0 (0)	11 (25)	11 (25)
	Total	7(16)	24 (53)	13 (30)	44 (100)

RDCT = regular-dose CT, ULDCT = ultralow-dose CT.

**Table 3.** Crosstab for nodule volume classification

	RDCT				
ULDCT	Volume (mm^3^)	<100	100 to <300	≥300	Total
	<100	21(47)	2 (4)	0 (0)	23 (51)
	100 to <300	0 (0)	11 (24)	0 (0)	11 (24)
	≥300	0 (0)	0 (0)	10 (23)	10 (23)
	Total	21 (47)	13 (29)	10 (23)	44 (100)

RDCT = regular-dose CT, ULDCT = ultralow-dose CT.

## Discussion

The purpose of this study was to investigate the detectability and diameter and volume quantification of pulmonary nodules in ULDCT compared to RDCT in a patient cohort with relatively high prevalence of lung nodules. Our study demonstrates comparable if not better detectability, with a sensitivity of 95.5% for ULDCT *vs* 88.6% for RDCT, at 83.1% reduced radiation dose, but with increased image noise (SD+13.8 HU). There was a statistically significant, but clinically irrelevant underestimation of nodule diameter and volume for ULDCT. This caused 4 (9.1%) and 2 (4.5%) of the nodules to be misclassified into lower size categories based on diameter and volume, respectively. Furthermore, for clinically relevant nodules, sized 30 mm^3^ or more on RDCT, the 95% LoA between ULDCT and the reference,RDCT, was similar (24.9%) compared to the clinically relevant 25% measurement error, used as cut-off to define nodule growth.^19^ Our findings suggest that semi-automatic volumetry on ULDCT with tin filter and ADMIRE could be used for serial imaging in lung cancer screening or in clinical setting, for detection and follow-up of lung nodules. The 95% LoA for semi-automatic diameter measurement was ± 1.4 mm, which is lower in measurement variability compared to the 95% LoA of ±1.7 mmin RDCT reported by Revel et al.^18^

A key feature in our study is the use of semi-automatic volumetry for lung nodule assessment on ULDCT. Benefits of volumetric measurements, such as reproducibility and sensitivity to detect nodule growth,^20^ has led to its support in lung cancer screening. Semi-automatic volumetry is currently the nodule assessment method recommended by European experts and the British Thoracic Society.^2,19^ Recently, Lung-RADS (v. 1.1) has included volume measurements next to diameter measurements to facilitate transition to 3D volume measurements over time.^21^ Although manual diameter measurement is still the most widely used method for nodule assessment, there is a gradual transition towards semi-automatic volumetry in lung cancer screening.

A couple of studies has evaluated the influence of major radiation dose reduction on lung nodule detection using submillisievert chest CT scans with IR.^22–27^ A previous phantom study evaluated third generation dual-source CT with ADMIRE for pulmonary nodule detection, with a scan protocol similar to ours.^9^ In their phantom study, Gordic et al found that ADMIRE strength levels 3 and 5 at 1/10th dose of a standard protocol offered similar sensitivity (94%) in nodule detection as compared to the standard protocol on the same CT system.^9^ Another study reported 91% sensitivity in nodule detection for ULDCT with ADMIRE level 3 compared to standard of reference in a diverse patient cohort.^16^ However, in their study, a different scanning protocol was used and the majority of participants, 169/202 (84%), underwent contrast-enhanced CT as reference method, which complicates comparison as iodine contrast enhancement impacts size measurements.^28^A recent phantom study by Janssen et al, using the same acquisition and ADMIRE strength as in this study,^25^ compared ULDCT to low-dose CT in nodule detection, and showed comparable sensitivity of 83% *vs* 84%, respectively. However, their study was limited by the use of an anthropomorphic phantom; with predetermined nodule size, shape and location. In our study, 50 patients were scanned with RDCT and ULDCT protocol. The readers were blinded from nodule information, and the RDCT and ULDCT scans were randomized and alternatively presented to reduce recall bias. More recently, Guo et al reported 93.6% sensitivity in nodule detection for ULDCT with ADMIRE level 3 in overweight or obese patients (BMI≥25 kg/m^2^), at 85% reduced radiation dose,^29^ which is similar compared to the sensitivity (95.5%) and radiation dose reduction (83%) in ULDCT in our study. This provides further evidence that ULDCT can be potentially used in a screening setting without being limited by screenee characteristics. In terms of nodule volumetry, two studies have reported slight underestimation of nodule volume in ULDCT when compared to RDCT, using comparable reconstruction settings as in our study.^30,31^ However, the underestimation was negligible in absolute volume, which is in line with our findings.

All patients included in our study were diagnosed with COPD. These patients often have architectural distortion with focal abnormalities and fibrosis on chest CT that can be difficult to distinguish from pulmonary nodules. Therefore, we used a consensus panel to review discrepant nodules found in RDCT and ULDCT. This is different compared to previous studies where two blinded readers reviewed the images independently, and discrepant results were discussed in a consensus meeting.^16,32,33^ Although consensus double reading can lead to more nodules being detected compared to single reading, it has been shown that consensus double reading does not offer substantial benefit in the detection of cancer, compared to single reading and a nodule management strategy based on semi-automatic volumetry.^34^

There are several strengths in our study. Firstly, we used a standardized RDCT scanning protocol, in line with the QIBA profile.^15^ Secondly, recall bias was avoided by blinding the readers from patient information and presenting the ULDCT and RDCT images in a random alternating order across several weeks. Thirdly, nodule diameters were measured semi-automatically by software, preventing inaccuracy from manual measurement. Manual diameter measurements in clinical practice are likely to be less precise and less accurate than the semi-automatic measurements used in our study. This has been shown in a previous study, which reported less variability and better nodule size classification based on Lung-RADS for semi-automatic diameter and volume measurements than for manual diameter measurements.^35^ This study has several limitations. Firstly, all participants in our study were known to have COPD. The difference between our study sample and a lung cancer screening population may limit the generalizability of our results. However, as the lung cancer screening population contains a much smaller proportion of COPD patients with severe lung abnormalities, we expect better sensitivity and less discrepancy in nodule detection. Moreover, studies have shown that patients with COPD have two- to five-fold greater lung cancer risk than smokers without COPD.^36,37^ Therefore, the result of our study is relevant for the lung cancer screening population. Thirdly, we did not evaluate the influence of ULDCT on the detection and quantification of subsolid nodules as these were not found in the participating patients. Fourthly, we used semi-automatic nodule measurement software of one vendor. Whether the results can be generalized to other types of semi-automatic software is uncertain. Lastly, our study is limited by its small sample size. However, this has not led to type II error, as small but significant differences were found between RDCT and ULDCT for both diameter and volume measurements.

ULDCT may not only prove itself useful in reducing radiation exposure for lung cancer screenings but may also be of benefit for the evaluation of emphysema in COPD patients outside the lung cancer screening setting. CT-based analyses can help quantify and differentiate phenotypes of COPD, which is crucial in determining appropriate management strategy.^38^ However, careful validation of ULDCT in the evaluation of emphysema is needed before utilization in the clinical practice.

In conclusion, at 83.1% reduced radiation dose, the nodule detectability in ULDCT remained comparable to RDCT. Agreement in size categories was good to excellent. Thus, the results shown in this study indicate that ULDCT with tin filter and ADMIRE may potentially be applicable in lung cancer screening.
